# A Two-Sample Analysis of the Construct Validity and Temporal Stability of a Process-Oriented Measure of Exercise Habit Strength

**DOI:** 10.21315/mjms-03-2025-169

**Published:** 2025-06-30

**Authors:** Hairul Anuar Hashim, Nur Hidayah Noordin, Syed Husin Syed Ahmad, Asmadi Ishak, Azizah Othman

**Affiliations:** 1 School of Health Sciences, Health Campus, Universiti Sains Malaysia, Kelantan, Malaysia; 2 Faculty of Health and Life Sciences, Management and Science University, Selangor, Malaysia; 3 Faculty of Sport Science and Coaching, Universiti Pendidikan Sultan Idris, Perak, Malaysia; 4 School of Medical Sciences, Health Campus, Universiti Sains Malaysia, Kelantan, Malaysia

**Keywords:** exercise habit, adolescents, obesity, psychometric properties, intraclass correlation coefficient

## Abstract

**Background:**

The Exercise Habit Strength Questionnaire (EHSQ) has shown excellent psychometric properties. However, children in different weight categories exhibit distinct patterns of exercise engagement. Therefore, examining the EHSQ’s psychometric properties across different weight groups across time may provide additional evidence of its discriminatory power and temporal stability.

**Methods:**

This study was conducted in two samples. Sample 1 involved 450 secondary school students (mean age: 14.72 ± 1.19 years), consisting of 52% males and 48% females across different body mass index (BMI) categories—normal (17.70%), overweight (23.44%), and obese (58.85%). Data collection involved the EHSQ, Body Appreciation Scale and the Implicit Association Test. Sample 2 consisted of 60 female students aged 16 years from all-girls schools, with 48.3% in the normal category and the remaining underweight, overweight, and obese categories. The EHSQ was administered twice, with a three-month interval between administrations.

**Results:**

Confirmatory factor analysis provided evidence of factorial validity (*χ*^2^ = 210.06, df = 129, *P* < 0.05; *χ*^2^/df = 1.63; CFI = 0.96; GFI = 0.95; RMSEA = 0.04; AIC = 314.69). Item loadings were above 0.5, except for one item, with latent factor intercorrelations ranging from 0.2 to 0.5. The results of the Intraclass correlation coefficient analysis demonstrated excellent test–retest reliability. Additionally, internal consistency scores in Sample 2 mirrored those of Sample 1, with Cronbach’s alpha values of 0.756 for patterned action, 0.757 for automaticity, 0.89 for negative consequences, and 0.84 for stimulus cue.

**Conclusion:**

Overall, the results provided adequate support for the hypothesised psychometric properties of the ESHQ.

## Introduction

The concept of exercise habit has garnered significant attention among researchers and practitioners in exercise psychology. This interest is driven by the growing prevalence of obesity and physical inactivity despite the well-documented health benefits of regular exercise (1). Identifying and measuring the processes of habit formation may offer a starting point for reversing this trend (2).

Grove et al. proposed several processes associated with the strength of habitual exercise behaviours (3, 4). First, these behaviours are characterised by a strong stimulus–response bond, where environmental cues can trigger exercise routines. Second, cue-driven behaviours are automatically initiated, requiring minimal conscious awareness and cognitive deliberation. Third, habitual exercise behaviours tend to follow a stable, consistent pattern, becoming increasingly resistant to change. Finally, the absence of exercise can lead to psychological distress if missed. Considerable evidence supports the role of these processes as viable indicators of habit strength (3, 4).

Research on exercise habits has largely been guided by social cognition theories, which assume that beliefs, attitudes, motives, and intentions influence decision-making processes and behaviours (5). Although substantial evidence supports the notion that these theories can explain physical activity behaviour, Rhodes and Dickau (6) suggested that incorporating non-conscious, implicit processes may better explain the intention–behaviour relationship.

Non-conscious processes can influence exercise behaviour in several ways. Habits, for example, are automatic behavioural responses triggered by associated cues developed through the repeated pairing of a cue with a specific behaviour (3, 7). Likewise, automatic evaluations—measured as implicit attitudes—reflect the strength of a person’s automatic association between a concept (e.g., physical activity) and feelings of pleasantness or unpleasantness. These evaluations can predispose individuals to respond to cues with approach or avoidance behaviours (8). Individuals with stronger exercise habits are more likely to have positive implicit attitudes toward exercise and physical activity (9).

Body appearance is frequently cited as a reason for exercise participation (10). One aspect of this is body appreciation, which refers to the unconditional acceptance of one’s body, rejecting stereotypical beauty standards and recognising its functionality and health (11). This positive body image has been linked to higher levels of exercise participation. Homan and Tylka (12) found that body appreciation among college women was positively associated with the frequency of physical activity, especially when the motivation was internalised (i.e., not driven by external factors). We hypothesised that stronger exercise habits are positively correlated with higher body appreciation.

An important consideration in understanding habit formation and evaluating interventions promoting regular physical activity is the availability of a valid and reliable measurement tool. In this regard, Grove and Zillich (3) developed the 18-item Exercise Habit Strength Questionnaire (EHSQ), which measures four subscales (automaticity, negative consequences, stimulus cue, and patterned action) representing the processes involved in habitual behaviour. Since its development, the EHSQ and its translated versions have demonstrated excellent psychometric properties.

Grove and Ortega (13) provided evidence of the EHSQ’s criterion validity in an adult sample. Hashim et al. (14) demonstrated the questionnaire’s factorial validity and internal consistency among adolescents aged 13 to 16 years in Western Australia. The Malaysian version also showed strong factorial validity and internal consistency in samples of children aged 10 to 11 years (15) and 13 to 16 years (16). Additionally, the EHSQ score exhibited a significant negative relationship (*r* = −0.24, *P* < 0.05) with 1-mile run times, providing further evidence of criterion validity in a sample of 10 to 11 years old. In a more recent study, the EHSQ demonstrated discriminatory patterns in hormonal and psychophysiological responses to different exercise intensities, further supporting its discriminant validity (17).

Although previous studies have supported the psychometric properties of the Malaysian version of the EHSQ, additional evidence is required for two main reasons. First, it has been shown that children in different weight categories exhibit distinct patterns of exercise engagement (18). Overweight and obese children generally have lower levels of physical activity compared to their normal weight peers. Therefore, examining the EHSQ’s psychometric properties across different weight groups may provide additional evidence of its discriminatory power. Second, no prior studies have established the temporal stability of the measure, which is essential for supporting its reliability over time.

Thus, the primary objective of this study was to investigate the construct validity of the EHSQ by examining its factorial structure and sensitivity in discriminating between known groups. Furthermore, this study aimed to provide evidence of temporal stability through a test–retest analysis. The hypotheses were as follows:

- Hypothesis 1: The EHSQ was expected to exhibit a four-factor structure.
- Hypothesis 2: The EHSQ was expected to demonstrate an excellent alpha coefficient.
- Hypothesis 3: EHSQ scores were expected to correlate negatively with body mass index (BMI) and positively with Body Appreciation Scale (BAS) scores.
- Hypothesis 4: EHSQ scores were expected to correlate positively with implicit attitude scores.
- Hypothesis 5: Individuals in different BMI categories were hypothesised to exhibit different exercise habit strength scores.
- Hypothesis 6: The EHSQ was expected to have a high intraclass correlation (ICC) in test–retest analysis.

## Methods

This two-sample, cross-sectional study was conducted from October 2022 to May 2023 in government secondary schools located in both the Eastern and Western regions of Malaysia. The analysis was based on two distinct samples: Sample 1 was used to examine the factorial validity and alpha reliability of the EHSQ, while Sample 2 was used to evaluate test–retest reliability, ICC, and predictive validity.

### Participants

#### Sample 1

The first sample consisted of 450 secondary school students (mean age = 14.72 ± 1.19 years), with 52% male and 48% female participants. The participants were classified into different BMI categories: normal weight (17.70%), overweight (23.44%), and obese (58.85%). The sample size exceeded the minimum required for factor analysis (i.e., 180 participants, following the recommendation of Tabachnick and Fidell which suggested at least 10 participants per questionnaire item) (19). The diversity in participants’ age and BMI categories was not expected to introduce significant bias, thereby ensuring the generalizability of the findings.

#### Sample 2

The second sample comprised of 60 female students, all aged 16 years, from all-girls’ secondary schools located in the Eastern region of Malaysia. These participants lived in dormitories and were classified as physically inactive, with no prior engagement in regular exercise programmes or habitual physical activity. The sample consisted of students across different BMI categories: underweight (26.7%), normal weight (48.3%), overweight (20.0%), and obese (5.0%). All participants were of Malay ethnicity.

### Instruments

#### Demographic Questionnaire

The first part of the questionnaire is related to demographic information, including age, sex, ethnicity, number of siblings, parents’ income, and location of residency. This section was used to screen participants for inclusion in the study.

#### Exercise Habit Strength Questionnaire

Habitual exercise behaviour was assessed using the 18-item EHSQ (1). This questionnaire comprises four subscales: automaticity, negative consequences for non-performance, stimulus cues, and patterned action. Example items include “I exercise without having to think about it” (Automaticity), “Certain surroundings just make me want to exercise” (Stimulus cues), and “If I don’t exercise, I feel restless” (Negative consequences). Responses were recorded on a 6-point Likert scale ranging from 1 (False) to 6 (True), with higher scores indicating stronger exercise habits.

#### Body Appreciation Scale-2

The BAS-2 (11) was used to measure participants’ body appreciation. This 10-item scale assesses body acceptance, respect for one’s body, and resistance to societal pressures regarding appearance. Items were rated on a 5-point Likert scale ranging from 1 (Never) to 5 (Always), yielding a total score between 5 and 50. Previous studies have demonstrated the scale’s high internal consistency (Cronbach’s *α* = 0.81–0.94) and convergent validity with self-esteem measures.

#### Physical Activity Enjoyment Scale (PACES)

A shortened version of PACES (20) was used to measure the enjoyment of physical activity. The 7-item scale used bipolar rating items (e.g., “I find it pleasurable” vs. “I find it unpleasurable”) to assess participants’ enjoyment of physical activity. Its validity and reliability have been well-established in previous research.

#### Implicit Association Test (IAT)

The IAT (21) was used to measure participants’ implicit attitudes toward physical activity and sedentary behaviour. The test involved categorising images of physical activity (e.g., jogging and playing sports) and sedentary behaviours (e.g., watching television and sitting at a computer) as either “active” or “sedentary,” while also evaluating the affective response (i.e., “good” or “bad”) to these stimuli. Data were collected using Inquisit Lab 6 software. To compute the IAT score, we replaced the top 10% fastest and slowest reaction times, calculated the difference between the average reaction times in the two critical blocks, and divided this by the pooled standard deviation (SD) of all latencies. This score ranged from −2 (strong implicit preference for sedentary behaviour) to +2 (strong implicit preference for physical activity), with 0 indicating no preference.

### Procedures

#### Sample 1

Following approval from the Ministry of Education, the District Education Office, school principals, and the University Research Ethics Committee, a briefing session was conducted for the students. During this session, the students were informed about the study’s nature, its objectives, the research procedures, potential benefits, and any potential risks associated with participation. It was emphasised that participation in the study was entirely voluntary, and students were free to withdraw at any time. At the conclusion of the briefing, an information pack containing detailed study information, an informed consent form for parents, and an assent form for the participants was distributed.

In a subsequent meeting, students who provided consent and received parental or legal guardian permission were given a set of questionnaires. This included the socio-demographic questionnaire, the EHSQ, the BAS-2, and participation in the IAT, as previously described. Participants’ height and weight were also measured during this session.

#### Sample 2

Data collection involved only the use of the EHSQ. Similar to Sample 1, a briefing session was conducted, during which students were informed about the nature of the study, its objectives, the research procedures, potential benefits, and possible risks. They were also informed that participation was voluntary, with the option to withdraw at any time. After the briefing, an information pack was distributed along with informed consent forms for their parents and assent forms for the participants. Once consent was obtained, participants completed the EHSQ at two timepoints: baseline and three months after the initial session. The administration of the questionnaire took place in the school dining hall, and a research assistant was present to assist with any queries.

### Data Analysis

#### Sample 1

For Sample 1, we employed several statistical techniques, including descriptive statistics, confirmatory factor analysis (CFA), Pearson correlation, and one-way multivariate analysis of variance (MANOVA). The model fit in CFA was assessed using the Chi-square statistic (*χ*^2^), with additional fit indices such as the root mean square residuals, where values < 0.05 indicated a close fit, and values up to 0.1 were considered acceptable. We also used the comparative fit index (CFI), with values ≥ 0.90 indicating a good fit. Additionally, the root mean square error of approximation (RMSEA) was used, with values ≤ 0.05 considered a close fit and values < 0.08 indicating acceptable fit (22). Data analysis was performed using IBM AMOS version 26 and IBM SPSS version 26.

#### Sample 2

For Sample 2, the ICC and Cronbach’s alpha were calculated to assess the internal consistency and temporal stability of the EHSQ and its subscales. All statistical computations were performed using IBM SPSS version 26.

## Results

### Sample 1: Construct Validity and Internal Consistency

The descriptive statistics for the primary measures from Sample 1 are provided in Tables 1 and 2. We tested the four-factor structure of the EHSQ, and the CFA results indicated a close model fit for the proposed structure (*χ*^2^ = 210.06, df = 129, *P* < 0.05; *χ*^2^/df = 1.63; CFI = 0.96; GFI = 0.95; RMSEA = 0.04; AIC = 314.69). Significant unstandardised regression weights (*P* < 0.05) were observed for all path loadings. Additionally, all standardised regression weights were ≥ 0.50, demonstrating convergent validity for the subscales.

Latent factor intercorrelations ranged from 0.20 to 0.50 across the subscales, with the following results: 0.50 (Pattern_Action ↔ Stimulus_Cue), 0.46, 0.50, 0.20, 0.45, and 0.35 for the relationships between the various subscales. Cronbach’s alpha values indicated acceptable internal consistency for pattern action (0.71), automaticity (0.65), negative consequences (0.76), and stimulus cue (0.78).

A higher-order model was also tested, yielding similarly favourable results (*χ*^2^ = 224.15, df = 131, *P* < 0.05; *χ*^2^/df = 1.71; CFI = 0.95; GFI = 0.95; RMSEA = 0.04; AIC = 304.15). Again, significant unstandardised regression weights (*P* < 0.05) and standardised regression weights above 0.50 were observed, further supporting the convergent validity of the subscale items.

Multigroup invariance analysis was conducted to compare the model across different BMI categories regarding the factor loadings of the EHSQ. No means or intercepts were estimated in these models. The unconstrained model fit was relatively weak, though acceptable (*χ*^2^ = 561.92, df = 387, *χ*^2^/df = 1.45, *P* < 0.001; GFI = 0.88; CFI = 0.90; RMSEA = 0.03; AIC = 813.92). No significant difference was found between the constrained and unconstrained models, suggesting invariance across BMI groups (Δ*χ*^2^ (28) = 27.760, *P* > 0.05). As in previous analyses, significant unstandardised regression weights (*P* < 0.05) and standardised regression weights above 0.50 were observed, confirming the convergent validity of the subscales.

Using one-way MANOVA, total EHSQ and subscale scores were compared across different BMI categories. No significant differences were found in EHSQ scores between normal, overweight, and obese adolescents (*F* = 1.302, df = 8.000, *P* = 0.239).

Correlation analysis revealed a significant negative relationship between EHSQ total scores and BMI (*r* = −0.10, *P* < 0.05), supporting the hypothesised association. Additionally, a significant positive correlation was found between exercise habit strength and BAS-2 scores (*r* = 0.25, *P* < 0.05). No significant relationship was observed between implicit attitude and habit strength scores. Further correlation values are presented in Table 3.

### Sample 2: Test–retest Reliability and Internal Consistency

Table 4 presents the results of the ICC analysis for Sample 2. Overall, the ICC values indicated excellent test–retest reliability. The internal consistency of the EHSQ in Sample 2 closely mirrored that of Sample 1, with Cronbach’s alpha values of 0.756 for patterned action, 0.757 for automaticity, 0.89 for negative consequences, and 0.84 for stimulus cue.

## Discussion

Our study aimed to provide evidence for the construct validity and internal consistency of the EHSQ in Sample 1. Factorial validity, a key aspect of construct validity, is demonstrated when the data aligns with the proposed factor structure of the questionnaire. Consistent with previous research, we found that the EHSQ’s four-factor structure was supported, reinforcing its conceptual framework (2, 4). The items were hypothesised to converge onto their respective factors, with the factors themselves exhibiting discriminant validity, meaning they should not correlate highly with one another. Our results confirmed this hypothesis, as the standardised factor loadings exceeded 0.5 for all items, and the latent factor intercorrelations ranged between 0.2 and 0.5, further supporting the discriminant validity of the subscales.

Additionally, multigroup invariance analysis revealed measurement invariance across different BMI categories, indicating that the factorial structure of the EHSQ was interpreted consistently among adolescents of varying weight groups (23). This psychometric quality enhances the questionnaire’s robustness, suggesting that the EHSQ can be reliably used across populations with different weight classifications.

A valid psychometric tool should also differentiate scores between groups with established differences, such as normal weight versus obese groups. McManus et al. reviewed physical activity behaviours and found that obese children tend to engage in lower levels of physical activity, regardless of the method of assessment (18). While our study observed lower EHSQ scores for overweight and obese adolescents, the differences in total exercise habit scores and subscale scores were insignificant, inconsistent with previous findings.

A plausible explanation for this inconsistency can be drawn from Graf et al. who found that despite physical and motor ability differences between obese and normal children, their leisure habits did not differ (24). This may suggest that while physical activity is typically measured by frequency and amount, exercise habit is a more process-oriented construct. Habit formation involves the repeated activation of behaviour by environmental cues, meaning that physical activity may occur without being habitual. Therefore, the lack of significant differences in exercise habit strength may be explained by the distinction between physical activity behaviour and exercise habit.

Despite the lack of significant differences between BMI categories, we did observe a significant negative relationship between BMI and exercise habit strength. This warrants further investigation into the relationship between habit strength and BMI among adolescents. Another key aspect of a valid questionnaire is its ability to demonstrate convergent and discriminant validity, which can be established by correlating it with constructs that are similar (convergent validity) or different (discriminant validity) (25). In our study, we hypothesised that exercise habit strength would positively correlate with body appreciation, negatively correlate with BMI, and positively correlate with implicit attitudes.

Overall, the results were mixed. EHSQ total and subscale scores were positively correlated with BAS-2 scores, supporting the hypothesis. Moreover, total EHSQ scores were significantly and negatively correlated with BMI. Although the subscale correlations with BMI were insignificant, they still exhibited a negative trend. Interestingly, the total and subscale scores of the EHSQ were not significantly correlated with implicit attitude scores, though the trend of the relationships was also negative.

The role of implicit attitude in influencing exercise behaviours has produced mixed results in the literature. While implicit attitude is thought to play a unique role in driving exercise behaviour, studies by Conroy et al. and Hyde et al. found no significant relationship between implicit attitudes and physical activity behaviours (26, 27). Rebar et al. proposed that the relationship between non-conscious regulatory processes and physical activity may be indirect, mediated by factors such as explicit attitudes and beliefs, which may explain our findings (28).

Finally, test–retest reliability is essential to confirm that a questionnaire’s scores remain stable over time. Habit, by definition, is a stable construct resistant to change (29). Our study provided strong evidence of the EHSQ’s stability, with excellent test–retest reliability over a three-month period. While this time frame represents a relatively short-term evaluation, we recommend that future studies assess the questionnaire’s stability over longer intervals.

## Conclusion

Altogether, our analyses from both samples partially supported the ESHQ’s construct validity. While we could not detect a significant difference in exercise habit scores among different BMI categories, we observed a significant negative relationship between the two variables. Future research should investigate this aspect and the insignificant relationship between implicit attitudes and exercise habit strength scores. In practical terms, we believe that the ESHQ may be a valid and reliable measure of habit strength and may also detect changes in processes associated with habitual behaviours.

## Figures and Tables

**Table 1:** Descriptive statistics of the participants’ background

	*N*	Min	Max	Mean	SD	Skewness		Kurtosis	
	
						Statistic	Std. error	Statistic	Std. error
Age	418	13.00	17.00	14.77	1.180	0.18	0.12	−0.81	0.24
Weight	418	33.00	146.00	75.33	17.530	0.20	0.12	0.58	0.24
Height	418	1.32	1.88	1.62	0.084	0.01	0.12	0.44	0.24
BMI	418	16.20	48.90	28.49	5.590	0.08	0.12	0.15	0.24

**Table 2:** Descriptive statistics of the primary measures

		*N*	Mean	SD	Std. error	95% confidence interval for mean		Min	Max

						Lower bound	Upper bound		
Patterned Action	Normal	74	17.40	5.69	0.66	16.08	18.71	5.00	30.00
	Overweight	98	16.98	6.48	0.65	15.68	18.28	5.00	29.00
	Obese	246	16.90	5.87	0.37	16.17	17.64	5.00	29.00
	Total	418	17.01	5.98	0.29	16.43	17.58	5.00	30.00
Automaticity	Normal	74	15.01	5.02	0.58	13.85	16.17	4.00	24.00
	Overweight	98	14.68	4.85	0.49	13.71	15.65	4.00	24.00
	Obese	246	14.70	4.45	0.28	14.14	15.25	4.00	24.00
	Total	418	14.75	4.64	0.23	14.30	15.19	4.00	24.00
Negative consequences for non-performance	Normal	74	13.38	6.03	0.70	11.99	14.78	5.00	30.00
	Overweight	98	12.01	5.83	0.59	10.84	13.18	5.00	30.00
	Obese	246	12.67	5.38	0.34	11.99	13.34	5.00	27.00
	Total	418	12.64	5.61	0.27	12.10	13.18	5.00	30.00
Stimulus cue	Normal	74	16.97	4.78	0.56	15.86	18.08	6.00	24.00
	Overweight	98	16.95	4.90	0.50	15.97	17.94	4.00	24.00
	Obese	246	16.32	4.84	0.31	15.71	16.93	4.00	24.00
	Total	418	16.58	4.84	0.24	16.12	17.05	4.00	24.00
Habit strength scores	Normal	74	62.76	14.37	1.67	59.43	66.09	22.00	91.00
	Overweight	98	60.63	16.42	1.66	57.33	63.92	21.00	100.00
	Obese	246	60.59	14.73	0.94	58.74	62.44	21.00	97.00
	Total	418	60.98	15.07	0.74	59.53	62.43	21.00	100.00
Body appreciation	Normal	74	37.80	8.23	0.96	35.89	39.70	18.00	50.00
	Overweight	98	35.79	9.00	0.91	33.98	37.59	13.00	50.00
	Obese	246	33.30	8.10	0.52	32.29	34.32	12.00	50.00
	Total	418	34.68	8.51	0.42	33.86	35.50	12.00	50.00
Explicit attitude	Normal	74	4.04	0.76	0.09	3.86	4.22	2.00	5.00
	Overweight	98	3.76	0.86	0.09	3.59	3.94	1.38	5.00
	Obese	246	3.85	0.81	0.05	3.75	3.95	1.75	5.00
	Total	418	3.86	0.82	0.04	3.79	3.94	1.38	5.00
Implicit attitude (D-score)	Normal	74	0.94	0.30	0.03	0.87	1.00	0.07	1.37
	Overweight	98	0.91	0.30	0.03	0.84	0.97	−0.24	1.44
	Obese	246	0.89	0.31	0.02	0.85	0.93	−0.15	1.54
	Total	418	0.90	0.31	0.01	0.87	0.93	−0.24	1.54

**Table 3:** Correlation matrix among the primary measures

			1	2	3	4	5	6	7	8	9
1.	BMI	*r*	1	−0.07	−0.06	−0.07	−0.10^*^	−0.10^*^	−0.23^**^	−0.13^**^	−0.08
		*P*		0.15	0.26	0.16	0.05	0.05	< 0.001	0.01	0.11
2.	Patterned action	*r*	−0.070	1	0.35^**^	0.40^**^	0.35^**^	0.76^**^	0.24^**^	0.36^**^	0.07
		*P*	0.15		< 0.001	< 0.001	< 0.001	< 0.001	< 0.001	< 0.001	0.14
3.	Automaticity	*r*	−0.06	0.35^**^	1	0.22^**^	0.41^**^	0.66^**^	0.11^*^	0.30^**^	0.07
		*P*	0.26	< 0.001		< 0.001	< 0.001	< 0.001	0.02	< 0.001	0.18
4.	Negative consequences for non-performance	*r*	−0.07	0.40^**^	0.22^**^	1	0.35^**^	0.71^**^	0.13^**^	0.23^**^	0.03
		*P*	0.16	< 0.001	< 0.001		< 0.001	< 0.001	0.01	< 0.001	0.60
5.	Stimulus cue	*r*	−0.10*	0.35^**^	0.41^**^	0.35^**^	1	0.72^**^	0.22^**^	0.41^**^	0.05
		*P*	0.05	< 0.001	< 0.001	< 0.001		< 0.001	< 0.001	< 0.001	0.27
6.	Habit strength scores	*r*	−0.10^*^	0.76^**^	0.66^**^	0.71^**^	0.72^**^	1	0.25^**^	0.45^**^	0.08
		*P*	0.04	< 0.001	< 0.001	< 0.001	< 0.001		< 0.001	< 0.001	0.123
7.	Body appreciation	*r*	−0.23^**^	0.24^**^	0.11^*^	0.13^**^	0.22^**^	0.25^**^	1	0.30^**^	0.09
		*P*	< 0.001	< 0.001	0.02	0.01	< 0.001	< 0.001		< 0.001	0.08
8.	Explicit attitude	*r*	−0.13^**^	0.36^**^	0.30^**^	0.23^**^	0.41^**^	0.45^**^	0.30^**^	1	0.14^**^
		*P*	.007	< 0.001	< 0.001	< 0.001	< 0.001	< 0.001	< 0.001		0.01
9.	Implicit attitude (D-Score)	*r*	−0.08	0.07	0.07	0.03	0.05	0.08	0.09	0.14^**^	1
		*P*	0.11	0.14	0.18	0.60	0.27	0.12	0.08	0.01	

**Table 4:** Intraclass correlation coefficient for the overall score and subscale scores

	Intraclass correlation	95% confidence interval		*F*-test with true value 0			
	
		Lower bound	Upper bound	Value	df1	df2	Sig
Patterned action	0.84	0.73	0.91	6.26	59	59	< 0.001
Automaticity	0.76	0.59	0.86	4.12	59	59	< 0.001
Negative consequences for non-performance	0.84	0.73	0.90	6.18	59	59	< 0.001
Stimulus cue	0.77	0.61	0.86	4.33	59	59	< 0.001
Overall	0.91	0.85	0.95	11.47	59	59	< 0.001
